# DIABETIC KETOACIDOSIS AS THE INITIAL PRESENTATION OF TYPE 1 DIABETES IN CHILDREN AND ADOLESCENTS: EPIDEMIOLOGICAL STUDY IN SOUTHERN BRAZIL

**DOI:** 10.1590/1984-0462/2020/38/2018204

**Published:** 2019-11-25

**Authors:** Leonardo Calil Vicente Franco de Souza, Gabriela de Carvalho Kraemer, Adriana Koliski, José Eduardo Carreiro, Mônica Nunes Lima Cat, Luiz De Lacerda, Suzana Nesi França

**Affiliations:** a Universidade Federal do Paraná. Curitiba, PR, Brazil

**Keywords:** Diabetes mellitus, Diabetes mellitus, type 1, Diabetic ketoacidosis, Diabetes melito, Diabetes melito tipo 1, Cetoacidose diabética

## Abstract

**Objective:**

To analyze the variables associated with the presence of diabetic ketoacidosis in type 1 diabetes mellitus (T1DM) diagnosis and its impact on the progression of the disease.

**Methods:**

We reviewed the records of 274 children and adolescents under 15 years, followed in a Pediatric Endocrinology clinic of a university hospital in Curitiba-PR. They had their first appointment between January 2005 and April 2015.

**Results:**

Most patients received their T1DM diagnosis during a diabetic ketoacidosis episode. The associated factors were: lower age and greater number of visits to a physician’s office prior to diagnosis; diabetic ketoacidosis was less frequent in patients who had siblings with T1DM and those diagnosed at the first appointment. Nausea and vomiting, abdominal pain, tachydyspnea, and altered level of consciousness were more common in the diabetic ketoacidosis group. There was no association with socioeconomic status, duration of symptoms before diagnosis, and length of the honeymoon period.

**Conclusions:**

Prospective studies are necessary to better define the impact of these factors on diagnosis and disease control. Campaigns to raise awareness among health professionals and the general population are essential to promote early diagnosis and proper treatment of diabetes mellitus in children and adolescents.

## RESUMO

**Objetivo:**

Avaliar as variáveis associadas ao diagnóstico de diabetes melito tipo 1 (DM1) na vigência de cetoacidose diabética e seu impacto na evolução da doença.

**Métodos:**

Foram avaliadas 274 crianças e adolescentes com idade até 15 anos acompanhados em um ambulatório de endocrinologia pediátrica de um hospital universitário de Curitiba, Paraná, cuja primeira consulta ocorreu entre janeiro de 2005 e abril de 2015.

**Resultados:**

A maioria dos pacientes teve diagnóstico de DM1 na vigência de cetoacidose diabética. Os fatores associados foram: menor idade e maior número de consultas prévias ao diagnóstico; a cetoacidose diabética foi menos frequente quando havia um irmão com DM1 e quando o diagnóstico foi feito na primeira consulta médica. Náuseas ou vômitos, dor abdominal, taquidispneia e alteração do nível de consciência foram mais frequentes no grupo com cetoacidose diabética ao diagnóstico. Não se observou associação com nível socioeconômico, tempo de sintomas antes do diagnóstico e duração do período de lua de mel.

**Conclusões:**

São necessários estudos prospectivos para definir melhor o impacto desses fatores no diagnóstico e no controle da doença. Campanhas de conscientização dos profissionais de saúde e da população são necessárias para que haja diagnóstico precoce e tratamento adequado do diabetes melito em crianças e adolescentes.

## INTRODUCTION

Type 1 diabetes mellitus (T1DM) is one of the most common chronic endocrine-metabolic diseases during childhood and adolescence, presenting with an uneven distribution among regions.^1^ Its worldwide incidence in children aged 15 years or younger is estimated at approximately 79,100 cases annually. In Brazil, the reported incidence is 10.4 cases of T1DM per 100,000 inhabitants.^1^ ^,^ ^2^

Diagnosis is based on criteria set by the American Diabetes Association (ADA) and the World Health Organization (WHO). The presence of autoantibodies associated with the destruction of pancreatic beta cells - anti-glutamic acid decarboxylase (anti-GAD), anti-tyrosine phosphatase (anti-IA2), anti-insulin (anti-IAA), anti-islet cells (anti-ICA), and anti-zinc transporter 8 (anti-ZnT8) - may corroborate the diagnosis, especially when there is some doubt about it.^2^

After the beginning of treatment with exogenous insulin, there may be a transitory phase, known as honeymoon, when insulin need is lower (under 0.5 UI/kg/day), and glycated hemoglobin (HbA1c) levels are under 7%. During this period, which can last from weeks to years and affects up to 80% of diabetic children and adolescents, a partial recovery of beta pancreatic cells function can occur, leading to increased insulin secretion and better peripheral sensibility.^2^

Diabetic ketoacidosis (DKA) is a complication of T1DM caused by insulin deficiency and the main cause of morbidity and mortality in children.^1^ The International Society for Pediatric and Adolescent Diabetes (ISPAD) defined the following diagnostic criteria for DKA: blood glucose above 200 mg/dL, metabolic acidosis (venous pH under 7.30 or serum bicarbonate under 15 mEq/L), and ketosis (ketonemia or ketonuria). DKA is classified, according to venous pH, into mild (under 7.30), moderate (under 7.20), or severe (under 7.10).^2^ Frequently, T1DM diagnosis is made during a DKA episode, which predicts worse metabolic control, including lower chances of a honeymoon period or having a shorter one, when it occurs.^2^ ^,^ ^3^ ^,^ ^4^

DKA at the start of T1DM might be more frequent on more aggressive forms of T1DM, with a sudden onset; or maybe family members and healthcare providers do not identify signs and symptoms previous to DKA.^5^ ^,^ ^6^ ^,^ ^7^ Thus, it is crucial to analyze this condition, evaluating possible risk factors for DKA presenting at T1DM diagnosis, and identifying delays in the diagnosis process.

The objectives of the present study were to evaluate the prevalence of DKA at T1DM diagnosis, identify factors associated with T1DM diagnosis during a DKA episode; and assess the impact of DKA at T1DM onset on disease control.

## METHOD

This is a cross-sectional study, based on the review of medical records of T1DM patients followed in the Pediatric Endocrinology outpatient clinic of *Hospital de Clínicas da Universidade Federal do Paraná* (HC-UFPR). The Ethics Committee of the institution approved the research project.

The list of patients was obtained from databases of the HC-UFPR informatics department and Pediatric Endocrinology clinic (*Unidade de Endocrinologia Pediátrica* - UEP). We analyzed charts of patients whose first appointment or first inpatient admission occurred between January 2005 and April 2015. Data related to social and demographic characteristics, diagnosis, disease progression, and follow-up in UEP were collected.

We distributed the patients into two groups, according to the initial presentation of T1DM. Patients from one group were diagnosed during a DKA episode, while the individuals from the other group had their diagnosis made without DKA. T1DM and DKA were defined and classified using criteria established by ISPAD.^2^ Patients diagnosed who started their treatment in other healthcare facilities than HC-UFPR were classified according to their chart information.

The present study excluded patients with a diabetes type other than 1, who had diabetes secondary to some other condition (for example, cystic fibrosis or chemotherapy), and those with uncertain diagnosis of T1DM. These individuals’ charts were not revised.

Among the 314 patients of the list, 40 were mistakenly included by the informatics department as they had no diagnosis of T1DM. Therefore, we reviewed 274 medical records. The sample size was calculated considering a 5% standard error, 10% type II error, 95% confidence interval, minimum sample mean difference of 10, and sample proportion of 15, providing the test with a 95% power.

The study evaluated the following variables: presence and severity of DKA at T1DM diagnosis, gender and age, socioeconomic characteristics (origin, household income, parents’ schooling, and family composition), frequency and duration of T1DM symptoms, number of medical visits before diagnosis, family history, laboratory tests (blood glucose, serum potassium, glycosuria, ketonuria, and autoantibodies), need for intensive care, frequency and duration of the honeymoon period, frequency of subsequent DKA episodes and hospitalizations, and comorbidities. The primary outcome of the study was the frequency of DKA, and the secondary ones were disease progression and complications.

Results were analyzed using the Statistica^®^ software. We compared the groups with the Pearson/Yates chi-square tests for categorical variables, and Student’s t-test, Mann-Whitney, and Kruskal-Wallis tests for continuous variables. During the analysis of each variable, we only considered existing information; excluding missing data in each analysis. A p value up to 0.05 was considered statistically significant.

## RESULTS

Out of 274 patients, 161 (58.8%) were diagnosed with T1DM during a DKA episode and 95 (34.7%) without DKA; 18 (6.6%) charts did not have information about the presence or absence of DKA at the time of T1DM diagnosis. It was possible to classify 83 of 161 DKA events at T1DM onset - 34 (41.0%) were mild, 20 (24.1%) were moderate, and 29 (34.9%) were severe.

Table 1 presents the general sample characteristics. Age at diagnosis varied between 0.8 and 14.6 years, with a median of 7.8 years and a difference among patients with and without DKA (median age of 7.0 and 8.3 years respectively, p<0.01). In the DKA group, 34.2% of the patients were under 5 years, and in the other group, 17.9% (p<0.01).

**Table 1:** General sample characteristics.

	TOTAL (n=274)*	DKA (n=161)	NO DKA (n=95)	p-value
Gender (%)				
Male	49.3	50.3	47.4	0.740
Female	50.7	49.7	52.6	
Origin (%)				
Curitiba	34.2	32.3	38.4	0.480
Metropolitan area	39.4	41.6	37.4	
Interior of Paraná (urban area)	17.9	18.0	16.5	
Interior of Paraná (rural area)	7.4	6.8	6.6	
Other Brazilian States	1.1	1.3	1.1	
Age at diagnosis (years)				
Minimum	0.8	0.8	1.0	<0.001
Maximum	14.6	14.6	14.1	
Median	7.8	7.0	8.3	

*n=274 for gender; n=269 for origin; n=273 for age at diagnosis. DKA: diabetic ketoacidosis.

Per capita household income ranged from 0.07 to 1.97 times the minimum wage, with a median of 0.5. The income of the groups with or without DKA did not present a significant difference (p=0.19). Most of the parents of the patients had not completed elementary school (35.9% of fathers, 30.1% of mothers) or had completed only high school (35.9% of fathers, 37.5% of mothers), and just a small portion went to college. Most patients (71.4%) lived with both parents at the moment of the first appointment. Parents’ schooling and family structure did not differ between groups.

Table 2 summarizes the frequency of signs and symptoms at diagnosis. Median time from the beginning of symptomatology until T1DM diagnosis was similar (p=0.90), 15 and 14 days, respectively, in groups with and without DKA.

**Table 2:** Symptoms presented prior to diagnosis.

	Total (n=245)	DKA (n=161)	No DKA (n=95)	p-value
Polydipsia (%)	92.7	91.4	94.5	0.53
Polyuria (%)	90.2	90.0	90.1	0.84
Weight loss or impaired weight gain (%)	73.2	74.3	71.4	0.74
Nauseas or vomiting (%)	29.7	43.6	9.9	<0.001
Polyphagia (%)	24.8	24.3	23.1	0.95
Abdominal pain (%)	23.6	32.1	13.2	0.001
Nocturia or enuresis (%)	22.8	21.4	27.5	0.37
Asthenia (%)	20.7	24.3	16.5	0.21
Tachypnea or dyspnea (%)	7.3	12.9	0.0	<0.001
Hyporexia (%)	5.7	8.6	2.2	0.88
Impaired consciousness (%)	4.9	8.6	0.0	0.01
Other (%)	20.3	26.4	13.2	0.26

DKA: diabetic ketoacidosis.

The DKA group showed a greater number of medical visits before diagnosis (p<0.001). Out of the 75 patients whose charts had some information about previous diagnoses, 38 were correctly diagnosed with T1DM at the first appointment; this finding was more prevalent among those who did not present DKA at T1DM onset (p<0.001). Table 3 lists other diagnoses made. One patient might have received more than one diagnosis before T1DM.

**Table 3:** Differential diagnoses of type 1 diabetes mellitus made in previous medical visits.

	Number of patients (n=37)
Gastroenteritis	11
Urinary tract infection	7
Bacterial tonsillitis	5
Common cold	4
Bronchopneumonia	4
Intestinal parasitosis	2
Oral or perineal candidiasis	2
Acute asthma attack	2
Psychosomatic disorder	1
Intentional weigh loss	1
Acute appendicitis	1
Non-specified gastric disorder	1
H1N1 flu	1
Acute rhinosinusitis	1

Note: Some patients had received more than one diagnosis before type 1 diabetes mellitus was detected.

In the sample, the percentage of relatives with T1DM was: 1.8% of fathers, 1.1% of mothers, 4.4% of siblings. A sibling previously diagnosed with T1DM was more frequent in the group diagnosed without DKA when compared to the group with DKA at T1DM diagnosis (10.5 and 1.2%, respectively; p=0.02).

We also analyzed laboratory tests. The DKA group had higher blood glucose levels in comparison to the group diagnosed before DKA (median of 448.0 and 350.5 mg/dL, respectively; p<0.001). Serum potassium and frequency of glycosuria and ketonuria showed no statistic difference.

Autoantibodies were investigated in 113 patients. The frequency of positivity was: 56.8% for anti-GAD, 44.8% for anti-IA2, 43.7% for anti-ICA, and 26.3% for anti-IAA. We found no relationship between autoantibodies and DKA at first manifestation of T1DM.

At diagnosis, 51.0% of patients were transferred to an intensive care unit (ICU), some due to severe clinical presentation, others to follow management protocols. Analyzing the DKA group, during ICU stay, 27.3% needed mechanical ventilation and 59.1% did not; while 13.6% were not transferred to ICU. In the non-DKA group, 7.8% were admitted to ICU without mechanical ventilation, and 92.2% did not need intensive care (p<0.001).

Seven patients developed complications caused by DKA at the moment of T1DM diagnosis: cerebral edema (2 patients), ischemic stroke (1 patient), cardiopulmonary arrest (1 patient), cardiac arrhythmia (1 patient), myocardial dysfunction (1 patient), and diabetes insipidus due to cerebral sinus thrombosis and infarction (1 patient).

In 95 cases, it was possible to define the beginning of the honeymoon period, and 9 of these patients were at this phase when we analyzed their medical records. From the moment of diagnosis to the start of the honeymoon, the median was 7.4 weeks, without statistic difference between groups (p=1.00). Among those whose honeymoon period had ended, the median duration was 6.1 months, also with no difference between groups (p=0.29).

Out of the 274 studied patients, 62 had new DKA episodes after starting insulin therapy, with a total of 106 hospitalizations. Among them, 27 patients were responsible for 71 hospitalizations, and 1 was admitted another 10 times with DKA after the beginning of treatment. The number of hospitalizations for subsequent DKA events ranged from 0 to 4 in the group diagnosed with T1DM without DKA, and from 0 to 10 in the group diagnosed with DKA, but the difference between groups was not significant. Other causes of hospitalizations after T1DM diagnosis included 29 patients admitted for hyperglycemia without DKA and 14 for hypoglycemia. Other causes of hospitalization included: urinary tract infection, pneumonia, acute gastroenteritis, seizure without hypoglycemia, acute asthma attack, meningitis, surgical procedure, among others. The incidence of hospitalizations for these causes was the same in both groups.

During outpatient follow-up, autoimmune diseases were detected - hypothyroidism (12.8%), celiac disease (2.9%), vitiligo (1.1%), psoriasis (0.4%), and alopecia areata (0.4%). Only two patients presented a chronic complication of T1DM during follow-up - one with neuropathy, and the other with nephropathy. Other associated disorders included: atopy (14.2%), dyslipidemia (3.6%), obesity or overweight (2.5%), epilepsy (2.5%), depression (1.8%), congenital heart disease (1.5%), attention deficit and hyperactivity disorder (1.1%), and hemangioma (0.7%); for this evaluation, we only considered conditions presented by more than one patient. The groups showed no statistic difference regarding patient comorbidity.

The median follow-up time in UEP was 3.4 years. Out of the 274 patients, 139 were being followed in UEP when their charts were reviewed, 63 were transferred to a transition clinic, and 71 did not come back or were being treated at another healthcare service. One patient died during the follow-up period due to a DKA episode that progressed to cerebral edema, venous sinus thrombosis, and secondary venous infarction, followed by brain death.

Using multivariate logistic regression, and considering the presence of DKA at T1DM diagnosis as the dependent variable, and age at diagnosis, household income, family history, and parents’ schooling as independent variables, the age at diagnosis increased 3 times the risk of DKA as initial presentation (*Odds Ratio* - OR 2.82; confidence interval of 95% - 95%CI 1.32‒6.04), while having a sibling with T1DM was a protective factor (OR 0.11; 95%CI 0.02‒0.52).

## DISCUSSION

Frequency of DKA at diagnosis varies among regions, occurring in 15‒70% of cases in Europe and North America.^2^ Brazilian studies have high incidences of DKA as the main manifestation at diagnosis: 67% in São Paulo^8^ and 42.3% in a Brazilian multicenter study.^9^ The present study shows high rates of DKA at T1DM onset. Although consistent with the Brazilian literature, this fact is possibly due to failures in the medical care of patients with symptoms that suggest T1DM.

Factors associated with DKA at T1DM onset are: age under five years (especially under two years), lower socioeconomic status, delay in T1DM diagnosis, and residence in countries with a low prevalence of T1DM. A higher prevalence of T1DM would lead to a greater local awareness of relatives and health professionals, anticipating diagnosis and the beginning of treatment. On the other hand, family history of T1DM is regarded as a protective factor for DKA at diagnosis.^2^ ^,^ ^5^ ^,^ ^10^ ^,^ ^11^

Previous studies indicate as possible risk factors for T1DM diagnosis made with DKA: lower household income and parents’ schooling. These factors could result in a worse understanding of symptoms, and also in limited access to healthcare services. The delay in seeking medical assistance, and, consequently, in reaching a T1DM diagnosis and starting treatment with insulin would allow the development of DKA.^2^ ^,^ ^10^ ^,^ ^12^ ^,^ ^13^ Patients followed in UEP are users of the Brazilian public health system, known as *Sistema Único de Saúde* (SUS), and most of them have low income and schooling. This might explain the lack of association of household income and parents’ schooling with DKA at diagnosis.

Children under 5 years, particularly those under 2‒3 years, are at higher risk of being diagnosed with T1DM in the presence of DKA, once symptoms like polyuria, polydipsia, and polyphagia, are harder to identify in those wearing diapers and breastfeeding. In the current study, the group with DKA at diagnosis showed lower median age than the other group; however, both had medians above five years, which should allow earlier diagnosis.^2^ ^,^ ^5^ ^,^ ^6^ ^,^ ^8^ ^,^ ^10^ ^,^ ^12^ ^,^ ^13^ ^,^ ^14^ ^,^ ^15^ ^,^ ^16^

Classic symptoms of T1DM include polydipsia and progressive weight loss, from 2 to 6 weeks. These were the most common symptoms found in the analyzed patients. Those with DKA had a higher frequency of nausea and vomiting, abdominal pain, tachypnea or dyspnea, and impaired consciousness, an expected result, as they are part of DKA clinical manifestations.^2^ ^,^ ^17^

Prior to T1DM diagnosis, the DKA group had a greater number of medical appointments, which could be an indication that DKA is the result of delayed diagnosis and start of insulin therapy. Nevertheless, intervals between symptoms and diagnosis were the same in both groups, and this might corroborate the hypothesis that DKA results from a more aggressive form of T1DM, with faster progress.

Theoretically, having a relative with T1DM would help parents recognize the symptoms, leading to earlier diagnosis, and being a protective factor for DKA.^2^ ^,^ ^15^ ^,^ ^16^ However, in this study, family history was only protective when one or more siblings had T1DM. Possibly, parents who already experienced the diagnosis of another son or daughter with T1DM recognize its manifestations more easily, while not always remembering the experience of their own diagnosis, which might have happened in childhood.

Differences in blood pH and serum bicarbonate, with statistical significance comparing both groups, were expected, once lower pH and bicarbonate are not only part of the DKA physiopathology, but also diagnostic criteria for this condition. In T1DM cases, serum potassium can decrease by osmotic diuresis and be even lower in DKA, due to secondary hyperaldosteronism caused by dehydration.^18^

Data about T1DM autoantibodies are limited, as these exams have been used more frequently only in recent T1DM cases. A Finish study revealed a lower frequency of DKA at T1DM onset in individuals who had anti-ZnT8,^19^ but this exam is not available in the hospital where the present research was held.

The great number of patients with DKA admitted to ICU was expected since this is part of the DKA management protocol in HC-UFPR, even in mild cases. The risk of cerebral edema and consciousness impairment in DKA and the need to preserve pervious airway might explain the higher prevalence of tracheal intubation and mechanical ventilation in these patients.^2^

Cerebral edema is a possible complication of DKA, and several hypotheses may explain it: neuronal dysfunction and edema, endothelial lesion followed by a rise in hydrostatic pressure during fluid administration, and high levels of antidiuretic hormone.^18^ A previous study conducted in our institution described 8 cases of cerebral edema due to DKA from a total of 327 hospitalizations, including T1DM new cases and children and adolescents diagnosed previously. Also, subtle and reversible manifestations were considered subclinical cerebral edema and observed in 21 cases.^18^ An Australian study explored how DKA at T1DM onset would affect cerebral functions, and revealed memory and attention impairments six months after DKA happened.^20^ DKA at T1DM diagnosis was also associated with changes in brain structure^21^ and lower cognitive functions^22^ in recent studies.

DKA as the initial presentation of T1DM is a predictor of worse glycemic control, including shorter honeymoon period and higher levels of HbA1c, although this result was not found in the present study.^2^ ^,^ ^3^ ^,^ ^4^ ^,^ ^23^

A small group of patients was responsible for most hospitalizations following DKA episodes, a fact already shown in previous studies.^2^ ^,^ ^18^ This finding can be related to different disease types, with greater susceptibility to DKA, or to more irregular treatment.

T1DM patients are predisposed to other autoimmune diseases, with higher prevalence when compared to the general population - hypothyroidism (3‒8%), hyperthyroidism (3‒6%), celiac disease (1‒10%), vitiligo (1‒7%), and primary adrenal insufficiency (2%).^2^ ^,^ ^24^ The present study showed a slightly higher prevalence of hypothyroidism than described in the literature. Only two patients had typical microvascular complications of T1DM. However, maybe the follow-up time was not long enough to evaluate it since patients are usually transferred to the adults’ clinic by the age of 15.

We noted a high proportion of lost follow-up, which might occur in several outpatient clinics of chronic diseases; sometimes due to parents’ lack of information or inappropriate living conditions, or because patients transferred their follow-up to another service without prior notification. We emphasize that, in UEP, the Social Service contacts patients with numerous absences only in cases of recent diagnosis.

A study held in the Italian province of Parma demonstrated that, after an educational campaign for parents, teachers, and physicians, rates of DKA at T1DM diagnosis decreased from 78 to 12.5%. Eight years later, another study was published, showing that the campaign had lasting effects, with DKA being the first recognized manifestation of T1DM in 15.6% of Parma diabetic children.^25^ ^,^ ^26^ Following this example, some other centers performed similar interventions, reducing the frequency of T1DM diagnosis made during a DKA episode.^27^

The main limitation of this study consists of it being based on past-recorded data, with some missing chart information. Nonetheless, we could identify factors associated with DKA at the time of T1DM diagnosis, and that some of them can be changed, especially the number of medical visits before T1DM is detected.

In conclusion, we found high rates of T1DM diagnosis with DKA. The main associated factor was lower age at disease onset. Although patients with DKA presented more intense symptoms and sought medical care more frequently, there was a delay in diagnosing them. DKA was less frequent in patients diagnosed in the first medical visit.

Prospective studies are necessary to better define the impact of these diagnostic factors on disease control. Awareness campaigns focused not only on health professionals but also on the general population should be made to achieve early diagnosis and appropriate treatment
